# Supplementary material for: Greater than pH 8: The pH dependence of EDTA as a preservative of high molecular weight DNA in biological samples
Source: PLoS One. 2023 Jan 23;18(1):e0280807. doi: 10.1371/journal.pone.0280807 (PMC9870144; doi:10.1371/journal.pone.0280807)
Supplement: S1 Table — Values are presented for tissues of Alitta virens, Faxonius virilis, Homarus americanus, Scomberomorus maculatus, and Mercenaria mercenaria that were either extracted immediately from fresh tissue or stored for 12 months in 0.25 M EDTA adjusted to pH 8, 9, or 10, or 95% ethanol. N/a indicates samples for which data were not collected; 95% EtOH, 95% ethanol; Fresh, untreated tissue extracted immediately after dissection; OGL, Ocean Genome Legacy Center specimen collection. (PDF) [file pone.0280807.s001.pdf]

OGL Specimen ID	Sample ID	Taxa	Treatment	Yield (µg)	Total Normalized Yield (µg DNA/mg tissue)	nY (µg DNA/mg tissue)	%R	*COI* PCR Amplification Success
S32141	MDW11	*Alitta virens*	Fresh	1.62	0.066942149	0.019132066	28.58	Yes
S32142	MDW12	*Alitta virens*	Fresh	1.8	0.064516129	0.036419355	56.45	Yes
S32143	MDW13	*Alitta virens*	Fresh	1.76	0.074576271	0.040099661	53.77	Yes
S32144	MDW14	*Alitta virens*	Fresh	5.05	0.206122449	0.111203061	53.95	Yes
S32145	MDW15	*Alitta virens*	Fresh	4.41	0.161538462	0.089621538	55.48	Yes
S32146	MDW16	*Alitta virens*	Fresh	3.87	0.16826087	0.089026826	52.91	Yes
S32147	MDW17	*Alitta virens*	Fresh	1.63	0.071179039	0.026072882	36.63	Yes
S32148	MDW18	*Alitta virens*	Fresh	1.77	0.055140187	0.025629159	46.48	Yes
S32149	MDW19	*Alitta virens*	Fresh	1.77	0.073443983	0.037640041	51.25	Yes
S32150	MDW20	*Alitta virens*	Fresh	2.69	0.09962963	0.053829889	54.03	Yes
S32141	MDW141	*Alitta virens*	EDTA pH 8	0.22	0.011340281	0.000628014	8.05	Yes
S32142	MDW142	*Alitta virens*	EDTA pH 8	0.7	0.031944444	0.0008	3.2	Yes
S32143	MDW143	*Alitta virens*	EDTA pH 8	0.215	0.009250531	0.0010105	10.81	Yes
S32144	MDW144	*Alitta virens*	EDTA pH 8	0.335	0.016600298	0.000635383	5.69	Yes
S32145	MDW145	*Alitta virens*	EDTA pH 8	1.06	0.04319507	0.000795	2.31	Yes
S32146	MDW146	*Alitta virens*	EDTA pH 8	0.188	0.008309029	0.000406266	6.98	Yes
S32147	MDW147	*Alitta virens*	EDTA pH 8	0.178	0.00498025	0.001222388	13.46	Yes
S32148	MDW148	*Alitta virens*	EDTA pH 8	0.251	0.00866905	0.000494331	7.09	Yes
S32149	MDW149	*Alitta virens*	EDTA pH 8	0.407	0.015417131	0.000792177	7.26	No
S32150	MDW150	*Alitta virens*	EDTA pH 8	0.361	0.012482427	0.000429113	4.41	Yes
S32141	MDW151	*Alitta virens*	EDTA pH 9	0.535	0.012680483	0.00077329	5.03	Yes
S32142	MDW152	*Alitta virens*	EDTA pH 9	4.92	0.145215927	0.017605522	9.59	Yes
S32143	MDW153	*Alitta virens*	EDTA pH 9	2.64	0.062460322	0.013122353	16.9	Yes
S32144	MDW154	*Alitta virens*	EDTA pH 9	2.75	0.090224275	0.011474702	14.02	Yes
S32145	MDW155	*Alitta virens*	EDTA pH 9	2.27	0.034733278	0.003843867	5.08	Yes
S32146	MDW156	*Alitta virens*	EDTA pH 9	0.549	0.00976048	0.002173988	16.79	Yes
S32147	MDW157	*Alitta virens*	EDTA pH 9	1.29	0.043152807	0.001604478	2.5	Yes
S32148	MDW158	*Alitta virens*	EDTA pH 9	1.39	0.038798443	0.001579746	3.58	Yes
S32149	MDW159	*Alitta virens*	EDTA pH 9	1.53	0.024705118	0.001516657	3.41	Yes
S32150	MDW160	*Alitta virens*	EDTA pH 9	2.13	0.104331102	0.003042857	2.8	Yes
S32141	MDW161	*Alitta virens*	EDTA pH 10	4.13	0.108305012	0.067514466	42.83	Yes
S32142	MDW162	*Alitta virens*	EDTA pH 10	4.55	0.108224016	0.066973214	57.7	Yes
S32143	MDW163	*Alitta virens*	EDTA pH 10	3.25	0.119399804	0.090217347	68.01	Yes
S32144	MDW164	*Alitta virens*	EDTA pH 10	1.64	0.095740249	0.053871713	82.45	Yes
S32145	MDW165	*Alitta virens*	EDTA pH 10	3.86	0.205855536	0.10253125	72.25	Yes
S32146	MDW166	*Alitta virens*	EDTA pH 10	1.74	0.077137168	0.041506025	76.81	Yes
S32147	MDW167	*Alitta virens*	EDTA pH 10	1.17	0.045703436	0.017823448	39.76	Yes
S32148	MDW168	*Alitta virens*	EDTA pH 10	3.03	0.115890755	0.072116322	62.12	Yes
S32149	MDW169	*Alitta virens*	EDTA pH 10	2.2	0.127484909	0.047728638	46.21	Yes
S32150	MDW170	*Alitta virens*	EDTA pH 10	5.83	0.227048212	0.150643454	64.34	Yes
S32141	MDW171	*Alitta virens*	95% EtOH	11.1	0.458233156	0.020161224	3.56	Yes
S32142	MDW172	*Alitta virens*	95% EtOH	9.19	0.30436262	0.019287167	4.89	Yes
S32143	MDW173	*Alitta virens*	95% EtOH	7.23	0.173055384	0.003831672	1.68	Yes
S32144	MDW174	*Alitta virens*	95% EtOH	9.07	0.229179013	0.013619772	4.61	Yes
S32145	MDW175	*Alitta virens*	95% EtOH	2.1	0.058393892	0.002178652	2.77	Yes
S32146	MDW176	*Alitta virens*	95% EtOH	6.92	0.223527085	0.058329957	19.64	Yes
S32147	MDW177	*Alitta virens*	95% EtOH	2.6	0.093596488	0.008160396	6.34	Yes
S32148	MDW178	*Alitta virens*	95% EtOH	2.64	0.091182091	0.002181818	2	Yes
S32149	MDW179	*Alitta virens*	95% EtOH	2.18	0.073774973	0.003370756	3.68	Yes
S32150	MDW180	*Alitta virens*	95% EtOH	4.77	0.165234375	0.01413	6.28	Yes
S32131	MDC11	*Faxonius virilis*	Fresh	0.275	0.01108871	0.003764617	33.95	Yes
S32132	MDC12	*Faxonius virilis*	Fresh	0.23	0.010454545	0.005044318	48.25	Yes
S32133	MDC13	*Faxonius virilis*	Fresh	0.33	0.012313433	0.00435403	35.36	Yes

OGL Specimen ID	Sample ID	Taxa	Treatment	Yield (µg)	Total Normalized Yield (µg DNA/mg tissue)	nY (µg DNA/mg tissue)	%R	*COI* PCR Amplification Success
S32134	MDC14	*Faxonius virilis*	Fresh	0.33	0.012359551	0.003400112	27.51	Yes
S32135	MDC15	*Faxonius virilis*	Fresh	0.361	0.016116071	0.006617259	41.06	Yes
S32136	MDC16	*Faxonius virilis*	Fresh	0.261	0.009255319	0.003346723	36.16	Yes
S32137	MDC17	*Faxonius virilis*	Fresh	0.334	0.012234432	0.0057563	47.05	Yes
S32138	MDC18	*Faxonius virilis*	Fresh	0.345	0.012873134	0.005523862	42.91	Yes
S32139	MDC19	*Faxonius virilis*	Fresh	0.635	0.02387218	0.010482274	43.91	Yes
S32140	MDC20	*Faxonius virilis*	Fresh	0.458	0.01647482	0.007835424	47.56	Yes
S32131	MDC141	*Faxonius virilis*	EDTA pH 8	3.4	0.066644929	0.033807774	28.14	Yes
S32132	MDC142	*Faxonius virilis*	EDTA pH 8	1.88	0.043966591	0.031157867	37.29	Yes
S32133	MDC143	*Faxonius virilis*	EDTA pH 8	3.93	0.095003149	0.055607054	34.1	Yes
S32134	MDC144	*Faxonius virilis*	EDTA pH 8	n/a	n/a	n/a	n/a	n/a
S32135	MDC145	*Faxonius virilis*	EDTA pH 8	3.92	0.050200848	0.059792099	49.42	Yes
S32136	MDC146	*Faxonius virilis*	EDTA pH 8	n/a	n/a	n/a	n/a	n/a
S32137	MDC147	*Faxonius virilis*	EDTA pH 8	0.802	0.009639423	0.002408498	9.64	Yes
S32138	MDC148	*Faxonius virilis*	EDTA pH 8	2.11	0.024823529	0.014670872	20.72	Yes
S32139	MDC149	*Faxonius virilis*	EDTA pH 8	4.41	0.048891353	0.051114234	41.61	Yes
S32140	MDC150	*Faxonius virilis*	EDTA pH 8	3.64	0.040088106	0.030571667	28.22	Yes
S32131	MDC151	*Faxonius virilis*	EDTA pH 9	1.94	0.044067659	0.056077906	80.07	Yes
S32132	MDC152	*Faxonius virilis*	EDTA pH 9	1.17	0.026323757	0.025861453	63.88	Yes
S32133	MDC153	*Faxonius virilis*	EDTA pH 9	4.45	0.112805041	0.120117986	75.04	Yes
S32134	MDC154	*Faxonius virilis*	EDTA pH 9	3.39	0.041140777	0.071632583	76.07	Yes
S32135	MDC155	*Faxonius virilis*	EDTA pH 9	0.528	0.013640832	0.015070957	65.65	Yes
S32136	MDC156	*Faxonius virilis*	EDTA pH 9	1.59	0.018047673	0.024172203	34.51	Yes
S32137	MDC157	*Faxonius virilis*	EDTA pH 9	0.661	0.015293892	0.021197274	70.23	Yes
S32138	MDC158	*Faxonius virilis*	EDTA pH 9	1.93	0.034123674	0.043330854	73.64	Yes
S32139	MDC159	*Faxonius virilis*	EDTA pH 9	0.987	0.014877668	0.0193452	67.62	Yes
S32140	MDC160	*Faxonius virilis*	EDTA pH 9	1.2	0.020291927	0.021224199	49.7	Yes
S32131	MDC161	*Faxonius virilis*	EDTA pH 10	0.556	0.01764992	0.017075196	62.65	Yes
S32132	MDC162	*Faxonius virilis*	EDTA pH 10	0.565	0.021565143	0.014965542	53.77	Yes
S32133	MDC163	*Faxonius virilis*	EDTA pH 10	0.534	0.020313813	0.010795459	52.36	Yes
S32134	MDC164	*Faxonius virilis*	EDTA pH 10	0.77	0.008369565	0.014483558	61.32	Yes
S32135	MDC165	*Faxonius virilis*	EDTA pH 10	2.65	0.040807255	0.054441385	60.81	Yes
S32136	MDC166	*Faxonius virilis*	EDTA pH 10	2.42	0.064060601	0.086954732	73.66	Yes
S32137	MDC167	*Faxonius virilis*	EDTA pH 10	0.726	0.011931102	0.013437849	58.86	Yes
S32138	MDC168	*Faxonius virilis*	EDTA pH 10	1.79	0.044516196	0.032559483	63.3	Yes
S32139	MDC169	*Faxonius virilis*	EDTA pH 10	0.324	0.008483196	0.005700183	46.27	Yes
S32140	MDC170	*Faxonius virilis*	EDTA pH 10	1.47	0.034831798	0.038794141	78.38	Yes
S32131	MDC171	*Faxonius virilis*	95% EtOH	3.23	0.082329029	0.042939275	34.83	Yes
S32132	MDC172	*Faxonius virilis*	95% EtOH	4.45	0.120150173	0.022832243	10.98	No
S32133	MDC173	*Faxonius virilis*	95% EtOH	1.78	0.049883898	0.031309082	36.41	Yes
S32134	MDC174	*Faxonius virilis*	95% EtOH	0.298	0.007341038	0.001700556	14.78	Yes
S32135	MDC175	*Faxonius virilis*	95% EtOH	6.99	0.137032481	0.032941964	12.96	Yes
S32136	MDC176	*Faxonius virilis*	95% EtOH	5.92	0.115892992	0.080784605	39.71	Yes
S32137	MDC177	*Faxonius virilis*	95% EtOH	1.14	0.03254468	0.036054066	57.56	Yes
S32138	MDC178	*Faxonius virilis*	95% EtOH	1.31	0.032621016	0.033800472	54.7	Yes
S32139	MDC179	*Faxonius virilis*	95% EtOH	4.38	0.097026932	0.067844938	37.64	Yes
S32140	MDC180	*Faxonius virilis*	95% EtOH	6.93	0.182634548	0.061210286	21.64	Yes
S32111	LSL1	*Homarus americanus*	Fresh	0.296	0.01034965	0.000198493	19.05	Yes
S32112	LSL2	*Homarus americanus*	Fresh	1.36	0.062100457	0.000218284	9.98	Yes
S32113	LSL3	*Homarus americanus*	Fresh	0.849	0.034096386	0.000180241	9.35	Yes
S32114	LSL4	*Homarus americanus*	Fresh	0.623	0.023246269	0.001414209	26.32	Yes
S32115	LSL5	*Homarus americanus*	Fresh	0.584	0.024232365	0.003932573	42.5	Yes
S32116	LSL6	*Homarus americanus*	Fresh	0.592	0.023399209	0.003369273	40.02	Yes

OGL Specimen ID	Sample ID	Taxa	Treatment	Yield (µg)	Total Normalized Yield (µg DNA/mg tissue)	nY (µg DNA/mg tissue)	%R	*COI* PCR Amplification Success
S32117	LSL7	*Homarus americanus*	Fresh	0.906	0.033932584	0.003616247	33.18	Yes
S32118	LSL8	*Homarus americanus*	Fresh	0.971	0.034432624	0.00491834	39.18	Yes
S32119	LSL9	*Homarus americanus*	Fresh	0.347	0.012664234	0.00028212	20.45	Yes
S32120	LSL10	*Homarus americanus*	Fresh	0.383	0.016872247	0.000435716	19.47	Yes
S32111	LSL131	*Homarus americanus*	EDTA pH 8	0.519	0.02595	0.00514761	51.22	Yes
S32112	LSL132	*Homarus americanus*	EDTA pH 8	0.23	0.008070175	0.0000216	13.5	Yes
S32113	LSL133	*Homarus americanus*	EDTA pH 8	0.969	0.042687225	1.83048E-05	3.92	Yes
S32114	LSL134	*Homarus americanus*	EDTA pH 8	0.82	0.03071161	0.000180178	8.14	Yes
S32115	LSL135	*Homarus americanus*	EDTA pH 8	0.924	0.033478261	0.006766435	47.52	Yes
S32116	LSL136	*Homarus americanus*	EDTA pH 8	1.02	0.045333333	0.0198536	74.7	Yes
S32117	LSL137	*Homarus americanus*	EDTA pH 8	0.317	0.014882629	0.000750473	23.37	Yes
S32118	LSL138	*Homarus americanus*	EDTA pH 8	0.904	0.031280277	0.007918028	55.14	Yes
S32119	LSL139	*Homarus americanus*	EDTA pH 8	0.533	0.018571429	5.47024E-05	8.82	Yes
S32120	LSL140	*Homarus americanus*	EDTA pH 8	0.497	0.017877698	0.000101364	11.84	Yes
S32111	LSL141	*Homarus americanus*	EDTA pH 9	0.217	0.009079498	0.001564117	52.8	Yes
S32112	LSL142	*Homarus americanus*	EDTA pH 9	0.475	0.017723881	4.57619E-05	6.89	Yes
S32113	LSL143	*Homarus americanus*	EDTA pH 9	0.594	0.023023256	0.000141996	8.62	Yes
S32114	LSL144	*Homarus americanus*	EDTA pH 9	1.03	0.039463602	0.012243579	58.42	Yes
S32115	LSL145	*Homarus americanus*	EDTA pH 9	0.26	0.009219858	7.48227E-05	16.88	Yes
S32116	LSL146	*Homarus americanus*	EDTA pH 9	0.257	0.010239044	0.003684538	65.59	Yes
S32117	LSL147	*Homarus americanus*	EDTA pH 9	0.271	0.012318182	0.002521309	51.36	Yes
S32118	LSL148	*Homarus americanus*	EDTA pH 9	0.519	0.019151292	0.006824756	69.27	Yes
S32119	LSL149	*Homarus americanus*	EDTA pH 9	0.239	0.01201005	0.000963742	29.46	Yes
S32120	LSL150	*Homarus americanus*	EDTA pH 9	0.375	0.018939394	0.000868169	23.71	Yes
S32111	LSL151	*Homarus americanus*	EDTA pH 10	0.262	0.008675497	0.000482861	29.76	Yes
S32112	LSL152	*Homarus americanus*	EDTA pH 10	0.874	0.039017857	0.010477938	59.57	Yes
S32113	LSL153	*Homarus americanus*	EDTA pH 10	0.611	0.024736842	0.009191429	66.97	Yes
S32114	LSL154	*Homarus americanus*	EDTA pH 10	0.538	0.017188498	0.004435137	60.62	Yes
S32115	LSL155	*Homarus americanus*	EDTA pH 10	0.29	0.011983471	0.002459417	50.87	Yes
S32116	LSL156	*Homarus americanus*	EDTA pH 10	0.313	0.010574324	0.002430456	53.29	Yes
S32117	LSL157	*Homarus americanus*	EDTA pH 10	0.241	0.008426573	0.001062659	40.74	Yes
S32118	LSL158	*Homarus americanus*	EDTA pH 10	0.404	0.012866242	0.004123701	64.42	Yes
S32119	LSL159	*Homarus americanus*	EDTA pH 10	0.3	0.012448133	0.00209271	46.27	Yes
S32120	LSL160	*Homarus americanus*	EDTA pH 10	0.362	0.014031008	0.004545767	64.44	Yes
S32111	LSL161	*Homarus americanus*	95% EtOH	4.37	0.197737557	0.003377158	17.52	Yes
S32112	LSL162	*Homarus americanus*	95% EtOH	3.63	0.146370968	0.004797714	19.41	Yes
S32113	LSL163	*Homarus americanus*	95% EtOH	1.28	0.050393701	0.000240034	8.14	Yes
S32114	LSL164	*Homarus americanus*	95% EtOH	0.622	0.021013514	0.001168919	27.68	Yes
S32115	LSL165	*Homarus americanus*	95% EtOH	4.01	0.131045752	0.017313333	44.52	Yes
S32116	LSL166	*Homarus americanus*	95% EtOH	2.09	0.114835165	0.000787088	11.46	Yes
S32117	LSL167	*Homarus americanus*	95% EtOH	1.06	0.034983498	0.000639343	16.01	Yes
S32118	LSL168	*Homarus americanus*	95% EtOH	1.66	0.079807692	0.004564423	47	Yes
S32119	LSL169	*Homarus americanus*	95% EtOH	1.42	0.04717608	0.000794791	21.36	Yes
S32120	LSL170	*Homarus americanus*	95% EtOH	0.491	0.019179688	0.000641305	19.78	Yes
S32101	MDF1	*Scomberomorus maculatus*	Fresh	0.817	0.035833333	0.00259075	7.23	
S32102	MDF2	*Scomberomorus maculatus*	Fresh	0.532	0.022931034	0.000605379	2.64	Yes
S32103	MDF3	*Scomberomorus maculatus*	Fresh	0.534	0.022820513	0.000844359	3.7	Yes
S32104	MDF4	*Scomberomorus maculatus*	Fresh	0.36	0.015450644	0.000633476	4.1	Yes
S32105	MDF5	*Scomberomorus maculatus*	Fresh	0.691	0.029656652	0.000940116	3.17	Yes
S32106	MDF6	*Scomberomorus maculatus*	Fresh	3.35	0.132936508	0.009784127	7.36	Yes
S32107	MDF7	*Scomberomorus maculatus*	Fresh	0.398	0.016048387	0.000908339	5.66	Yes
S32108	MDF8	*Scomberomorus maculatus*	Fresh	0.85	0.031135531	0.001628388	5.23	Yes
S32109	MDF9	*Scomberomorus maculatus*	Fresh	0.622	0.025387755	0.001414098	5.57	Yes

OGL Specimen ID	Sample ID	Taxa	Treatment	Yield (µg)	Total Normalized Yield (µg DNA/mg tissue)	nY (µg DNA/mg tissue)	%R	*COI* PCR Amplification Success
S32110	MDF10	*Scomberomorus maculatus*	Fresh	0.836	0.03512605	0.002680118	7.63	Yes
S32101	MDF131	*Scomberomorus maculatus*	EDTA pH 8	0.351	0.026811609	0.00128466	10.98	Yes
S32102	MDF132	*Scomberomorus maculatus*	EDTA pH 8	0.159	0.011128589	0.000617549	11.38	Yes
S32103	MDF133	*Scomberomorus maculatus*	EDTA pH 8	0.265	0.018617793	0.001101713	17.96	Yes
S32104	MDF134	*Scomberomorus maculatus*	EDTA pH 8	0.271	0.025468197	0.001075704	11.67	Yes
S32105	MDF135	*Scomberomorus maculatus*	EDTA pH 8	0.186	0.011030996	0.000999925	14.3	Yes
S32106	MDF136	*Scomberomorus maculatus*	EDTA pH 8	0.224	0.013214298	0.000788506	12.25	Yes
S32107	MDF137	*Scomberomorus maculatus*	EDTA pH 8	0.228	0.016080367	0.000924451	11.88	Yes
S32108	MDF138	*Scomberomorus maculatus*	EDTA pH 8	0.698	0.07088821	0.003309765	10.1	Yes
S32109	MDF139	*Scomberomorus maculatus*	EDTA pH 8	0.313	0.019088476	0.001200506	11.89	Yes
S32110	MDF140	*Scomberomorus maculatus*	EDTA pH 8	0.406	0.043381702	0.002707282	14.67	Yes
S32101	MDF141	*Scomberomorus maculatus*	EDTA pH 9	0.268	0.023894463	0.00149573	12.39	Yes
S32102	MDF142	*Scomberomorus maculatus*	EDTA pH 9	0.113	0.006636745	0.000190663	4.91	Yes
S32103	MDF143	*Scomberomorus maculatus*	EDTA pH 9	0.165	0.011286645	0.001077068	20.04	Yes
S32104	MDF144	*Scomberomorus maculatus*	EDTA pH 9	0.539	0.036865006	0.002821598	14.71	Yes
S32105	MDF145	*Scomberomorus maculatus*	EDTA pH 9	0.346	0.018659156	0.001572826	14.41	Yes
S32106	MDF146	*Scomberomorus maculatus*	EDTA pH 9	0.459	0.031092555	0.001539367	9.86	Yes
S32107	MDF147	*Scomberomorus maculatus*	EDTA pH 9	0.448	0.018637363	0.001192814	10.73	Yes
S32108	MDF148	*Scomberomorus maculatus*	EDTA pH 9	0.767	0.052824414	0.00525163	19.24	Yes
S32109	MDF149	*Scomberomorus maculatus*	EDTA pH 9	0.484	0.030419169	0.002239352	13.14	Yes
S32110	MDF150	*Scomberomorus maculatus*	EDTA pH 9	1.26	0.07428198	0.008484241	23.5	Yes
S32101	MDF151	*Scomberomorus maculatus*	EDTA pH 10	0.807	0.064941824	0.00606663	19.32	Yes
S32102	MDF152	*Scomberomorus maculatus*	EDTA pH 10	0.61	0.041012031	0.003456667	14.96	Yes
S32103	MDF153	*Scomberomorus maculatus*	EDTA pH 10	1.06	0.047620399	0.008183102	33.35	Yes
S32104	MDF154	*Scomberomorus maculatus*	EDTA pH 10	0.66	0.051097396	0.008496906	35.79	Yes
S32105	MDF155	*Scomberomorus maculatus*	EDTA pH 10	0.851	0.054084765	0.008367195	28.71	Yes
S32106	MDF156	*Scomberomorus maculatus*	EDTA pH 10	0.614	0.040349817	0.005380272	27.69	Yes
S32107	MDF157	*Scomberomorus maculatus*	EDTA pH 10	0.416	0.024546265	0.002192789	14.97	Yes
S32108	MDF158	*Scomberomorus maculatus*	EDTA pH 10	0.678	0.048836343	0.005363611	19.54	Yes
S32109	MDF159	*Scomberomorus maculatus*	EDTA pH 10	0.445	0.035449498	0.003437725	17.15	Yes
S32110	MDF160	*Scomberomorus maculatus*	EDTA pH 10	0.383	0.039170568	0.003706824	19.26	Yes
S32101	MDF161	*Scomberomorus maculatus*	95% EtOH	0.409	0.012200379	0.001957502	9.62	Yes
S32102	MDF162	*Scomberomorus maculatus*	95% EtOH	0.535	0.013525281	0.001192604	5.35	Yes
S32103	MDF163	*Scomberomorus maculatus*	95% EtOH	0.479	0.012613128	0.001128413	4.9	Yes
S32104	MDF164	*Scomberomorus maculatus*	95% EtOH	0.72	0.011944551	0.001875918	7.66	No
S32105	MDF165	*Scomberomorus maculatus*	95% EtOH	0.574	0.012260325	0.001166442	5.06	Yes
S32106	MDF166	*Scomberomorus maculatus*	95% EtOH	0.356	0.010377229	0.000941093	5.71	No
S32107	MDF167	*Scomberomorus maculatus*	95% EtOH	0.331	0.00790982	0.000864278	6.11	No
S32108	MDF168	*Scomberomorus maculatus*	95% EtOH	0.501	0.01379851	0.002876277	12.86	Yes
S32109	MDF169	*Scomberomorus maculatus*	95% EtOH	0.671	0.018846407	0.006448663	18.26	Yes
S32110	MDF170	*Scomberomorus maculatus*	95% EtOH	0.255	0.005530542	0.002986213	27.52	Yes
S32121	LSCL1	*Mercenaria mercenaria*	Fresh	9.22	0.364426877	0.252146957	69.19	Yes
S32122	LSCL2	*Mercenaria mercenaria*	Fresh	8.9	0.096529284	0.055456074	57.45	Yes
S32123	LSCL3	*Mercenaria mercenaria*	Fresh	7.33	0.313247863	0.154838419	49.43	Yes
S32124	LSCL4	*Mercenaria mercenaria*	Fresh	11.7	0.481481481	0.232892593	48.37	Yes
S32125	LSCL5	*Mercenaria mercenaria*	Fresh	8.83	0.314234875	0.183544591	58.41	Yes
S32126	LSCL6	*Mercenaria mercenaria*	Fresh	3.14	0.118045113	0.031600677	26.77	Yes
S32127	LSCL7	*Mercenaria mercenaria*	Fresh	4.28	0.157352941	0.092004265	58.47	Yes
S32128	LSCL8	*Mercenaria mercenaria*	Fresh	2.83	0.088993711	0.040545535	45.56	Yes
S32129	LSCL9	*Mercenaria mercenaria*	Fresh	11.2	0.405797101	0.196446377	48.41	Yes
S32130	LSCL10	*Mercenaria mercenaria*	Fresh	6.46	0.262601626	0.166620732	63.45	Yes
S32121	LSCL131	*Mercenaria mercenaria*	EDTA pH 8	3.23	0.110447038	0.084320717	61.87	Yes
S32122	LSCL132	*Mercenaria mercenaria*	EDTA pH 8	3.13	0.107494087	0.104096419	76.16	Yes

OGL Specimen ID	Sample ID	Taxa	Treatment	Yield (µg)	Total Normalized Yield (µg DNA/mg tissue)	nY (µg DNA/mg tissue)	%R	*COI* PCR Amplification Success
S32123	LSCL133	*Mercenaria mercenaria*	EDTA pH 8	4.13	0.137170161	0.144768252	86.23	Yes
S32124	LSCL134	*Mercenaria mercenaria*	EDTA pH 8	7.91	0.360758401	0.274100333	72.77	Yes
S32125	LSCL135	*Mercenaria mercenaria*	EDTA pH 8	6.44	0.290783055	0.238782634	76.01	Yes
S32126	LSCL136	*Mercenaria mercenaria*	EDTA pH 8	8.7	0.219448373	0.215260396	74.97	Yes
S32127	LSCL137	*Mercenaria mercenaria*	EDTA pH 8	8.33	0.332806366	0.266526138	78.71	Yes
S32128	LSCL138	*Mercenaria mercenaria*	EDTA pH 8	1.51	0.064737492	0.053791211	79.44	Yes
S32129	LSCL139	*Mercenaria mercenaria*	EDTA pH 8	1.7	0.087979497	0.059688889	75.84	Yes
S32130	LSCL140	*Mercenaria mercenaria*	EDTA pH 8	3.82	0.134568182	0.0961876	75.54	Yes
S32121	LSCL141	*Mercenaria mercenaria*	EDTA pH 9	3.16	0.120811373	0.08051551	74.91	Yes
S32122	LSCL142	*Mercenaria mercenaria*	EDTA pH 9	3.13	0.104779019	0.084993398	70.33	Yes
S32123	LSCL143	*Mercenaria mercenaria*	EDTA pH 9	2.64	0.102145166	0.070646829	65.83	Yes
S32124	LSCL144	*Mercenaria mercenaria*	EDTA pH 9	4.39	0.107746909	0.1020675	73.47	Yes
S32125	LSCL145	*Mercenaria mercenaria*	EDTA pH 9	6.57	0.196037187	0.175192601	78.93	Yes
S32126	LSCL146	*Mercenaria mercenaria*	EDTA pH 9	4.03	0.12979066	0.133701176	84.6	Yes
S32127	LSCL147	*Mercenaria mercenaria*	EDTA pH 9	7.05	0.234319792	0.252680472	83.51	Yes
S32128	LSCL148	*Mercenaria mercenaria*	EDTA pH 9	13.8	0.672740675	0.29156391	56.2	Yes
S32129	LSCL149	*Mercenaria mercenaria*	EDTA pH 9	3.93	0.209598795	0.17045089	82.84	Yes
S32130	LSCL150	*Mercenaria mercenaria*	EDTA pH 9	8.33	0.385749443	0.345359431	87.48	Yes
S32121	LSCL151	*Mercenaria mercenaria*	EDTA pH 10	0.892	0.046831497	0.028015258	69.41	Yes
S32122	LSCL152	*Mercenaria mercenaria*	EDTA pH 10	3.88	0.1753833	0.111541849	68.42	Yes
S32123	LSCL153	*Mercenaria mercenaria*	EDTA pH 10	2.18	0.068146101	0.05788058	73.28	Yes
S32124	LSCL154	*Mercenaria mercenaria*	EDTA pH 10	1.24	0.062868953	0.048608	76.44	Yes
S32125	LSCL155	*Mercenaria mercenaria*	EDTA pH 10	4.79	0.175345877	0.162899424	82.64	Yes
S32126	LSCL156	*Mercenaria mercenaria*	EDTA pH 10	0.862	0.025133074	0.026438258	73.61	Yes
S32127	LSCL157	*Mercenaria mercenaria*	EDTA pH 10	2.54	0.115534678	0.107618696	77.96	Yes
S32128	LSCL158	*Mercenaria mercenaria*	EDTA pH 10	6.37	0.272107843	0.179484118	76.64	Yes
S32129	LSCL159	*Mercenaria mercenaria*	EDTA pH 10	2.54	0.14745035	0.097507047	74.09	Yes
S32130	LSCL160	*Mercenaria mercenaria*	EDTA pH 10	7.16	0.199160502	0.218169412	88.06	Yes
S32121	LSCL161	*Mercenaria mercenaria*	95% EtOH	13.8	0.429054418	0.390021719	62.46	Yes
S32122	LSCL162	*Mercenaria mercenaria*	95% EtOH	17.6	0.403835729	0.465822951	64.58	Yes
S32123	LSCL163	*Mercenaria mercenaria*	95% EtOH	5.42	0.195287487	0.158031714	61.23	No
S32124	LSCL164	*Mercenaria mercenaria*	95% EtOH	5.21	0.12342751	0.160352222	74.79	Yes
S32125	LSCL165	*Mercenaria mercenaria*	95% EtOH	7.39	0.138846575	0.164828007	63.79	Yes
S32126	LSCL166	*Mercenaria mercenaria*	95% EtOH	8.69	0.2072218	0.237012201	70.64	Yes
S32127	LSCL167	*Mercenaria mercenaria*	95% EtOH	11.7	0.321933962	0.299445205	56.05	Yes
S32128	LSCL168	*Mercenaria mercenaria*	95% EtOH	2.17	0.054826026	0.051237354	68.71	Yes
S32129	LSCL169	*Mercenaria mercenaria*	95% EtOH	8.26	0.282031583	0.280995117	72.46	Yes
S32130	LSCL170	*Mercenaria mercenaria*	95% EtOH	9.65	0.242095554	0.223026346	60.09	Yes
