# Supplementary material for: Greater than pH 8: The pH dependence of EDTA as a preservative of high molecular weight DNA in biological samples
Source: PLoS One. 2023 Jan 23;18(1):e0280807. doi: 10.1371/journal.pone.0280807 (PMC9870144; doi:10.1371/journal.pone.0280807)
Supplement: S2 Table — The barcode region of the mitochondrial cytochrome oxidase I (COI) gene was sequenced from three specimens of each taxon used in this study. The values listed are percent identities to the best match found in the Barcode of Life Data System (BOLD) database. Specimen IDs for both the Ocean Genome Legacy Center online catalog and best matches found in BOLD are presented. N/a indicates samples for which sequencing was not successful; OGL, Ocean Genome Legacy Center specimen collection. (PDF) [file pone.0280807.s002.pdf]

OGL Specimen ID	Sample ID	Taxa	Treatment	BOLD Process ID	Species Identification of Best Match in BOLD	Sequence Identity (%)	Record ID of Best Match in BOLD
S32143	MDW13	*Alitta virens*	Fresh	EDTA036-21	*Alitta virens*	100	GBMNB36094-20
S32143	MDW163	*Alitta virens*	EDTA pH 10	EDTA037-21	*Alitta virens*	100	GBMNB36094-20
S32143	MDW173	*Alitta virens*	95% EtOH	EDTA038-21	*Alitta virens*	100	GBMNB36094-20
S32144	MDW14	*Alitta virens*	Fresh	EDTA039-21	*Alitta virens*	100	GBMNB36094-20
S32144	MDW164	*Alitta virens*	EDTA pH 10	EDTA040-21	*Alitta virens*	100	GBMNB36094-20
S32144	MDW174	*Alitta virens*	95% EtOH	EDTA041-21	*Alitta virens*	100	GBMNB36094-20
S32148	MDW18	*Alitta virens*	Fresh	EDTA044-21	*Alitta virens*	100	GBMNB36094-20
S32148	MDW168	*Alitta virens*	EDTA pH 10	EDTA042-21	*Alitta virens*	100	GBMNB36094-20
S32148	MDW178	*Alitta virens*	95% EtOH	EDTA043-21	*Alitta virens*	100	GBMNB36094-20
S32131	MDC11	*Faxonius virilis*	Fresh	EDTA010-21	*Orconectes virilis*	100	GBFCJ080-08
S32131	MDC161	*Faxonius virilis*	EDTA pH 10	EDTA011-21	*Orconectes virilis*	100	GBFCJ080-08
S32131	MDC177	*Faxonius virilis*	95% EtOH	EDTA015-21	*Orconectes virilis*	100	GBFCJ071-08
S32137	MDC17	*Faxonius virilis*	Fresh	EDTA014-21	*Orconectes virilis*	100	GBFCJ071-08
S32137	MDC167	*Faxonius virilis*	EDTA pH 10	EDTA013-21	*Orconectes virilis*	100	GBFCJ071-08
S32137	MDC171	*Faxonius virilis*	95% EtOH	EDTA012-21	*Orconectes virilis*	100	GBFCJ080-08
S32139	MDC19	*Faxonius virilis*	Fresh	EDTA018-21	*Orconectes virilis*	100	GBFCJ080-08
S32139	MDC169	*Faxonius virilis*	EDTA pH 10	EDTA016-21	*Orconectes virilis*	100	GBFCJ080-08
S32139	MDC179	*Faxonius virilis*	95% EtOH	EDTA017-21	*Orconectes virilis*	100	GBFCJ080-08
S32111	LSL1	*Homarus americanus*	Fresh	EDTA027-21	*Homarus americanus*	100	RBGC074-03
S32111	LSL151	*Homarus americanus*	EDTA pH 10	EDTA028-21	*Homarus americanus*	100	RBGC074-03
S32111	LSL161	*Homarus americanus*	95% EtOH	EDTA029-21	*Homarus americanus*	100	RBGC074-03
S32115	LSL5	*Homarus americanus*	Fresh	EDTA032-21	*Homarus americanus*	100	GBCM21816-19
S32115	LSL155	*Homarus americanus*	EDTA pH 10	EDTA030-21	*Homarus americanus*	100	GBCM21816-19
S32115	LSL165	*Homarus americanus*	95% EtOH	EDTA031-21	*Homarus americanus*	100	GBCM21816-19
S32117	LSL7	*Homarus americanus*	Fresh	EDTA035-21	*Homarus americanus*	100	GBCM21816-19
S32117	LSL157	*Homarus americanus*	EDTA pH 10	EDTA033-21	*Homarus americanus*	100	GBCM21816-19
S32117	LSL167	*Homarus americanus*	95% EtOH	EDTA034-21	*Homarus americanus*	100	GBCM21816-19
S32106	MDF6	*Scomberomorus maculatus*	Fresh	EDTA023-21	*Scomberomorus maculatus*	100	MFIV314-10
S32106	MDF156	*Scomberomorus maculatus*	EDTA pH 10	EDTA022-21	*Scomberomorus maculatus*	100	MFIV314-10
S32106	MDF166	*Scomberomorus maculatus*	95% EtOH	n/a	n/a	n/a	n/a
S32109	MDF9	*Scomberomorus maculatus*	Fresh	EDTA026-21	*Scomberomorus maculatus*	100	ANGBF41754-19
S32109	MDF159	*Scomberomorus maculatus*	EDTA pH 10	EDTA024-21	*Scomberomorus maculatus*	100	ANGBF41754-19
S32109	MDF169	*Scomberomorus maculatus*	95% EtOH	EDTA025-21	*Scomberomorus maculatus*	100	ANGBF41754-19
S32110	MDF10	*Scomberomorus maculatus*	Fresh	EDTA019-21	*Scomberomorus maculatus*	100	MFIV314-10
S32110	MDF160	*Scomberomorus maculatus*	EDTA pH 10	EDTA020-21	*Scomberomorus maculatus*	100	MFIV314-10
S32110	MDF170	*Scomberomorus maculatus*	95% EtOH	EDTA021-21	*Scomberomorus maculatus*	100	MFIV314-10
S32121	LSCL1	*Mercenaria mercenaria*	Fresh	EDTA001-21	*Mercenaria mercenaria*	100	GBML10502-19
S32121	LSCL151	*Mercenaria mercenaria*	EDTA pH 10	EDTA002-21	*Mercenaria mercenaria*	100	GBML10502-19
S32121	LSCL161	*Mercenaria mercenaria*	95% EtOH	EDTA003-21	*Mercenaria mercenaria*	100	GBML10502-19
S32124	LSCL4	*Mercenaria mercenaria*	Fresh	EDTA006-21	*Mercenaria mercenaria*	100	GBMLB2713-08
S32124	LSCL154	*Mercenaria mercenaria*	EDTA pH 10	EDTA004-21	*Mercenaria mercenaria*	100	GBMLB2713-08
S32124	LSCL164	*Mercenaria mercenaria*	95% EtOH	EDTA005-21	*Mercenaria mercenaria*	100	GBMLB2713-08
S32126	LSCL6	*Mercenaria mercenaria*	Fresh	EDTA009-21	*Mercenaria mercenaria*	100	GBML10502-19
S32126	LSCL156	*Mercenaria mercenaria*	EDTA pH 10	EDTA007-21	*Mercenaria mercenaria*	100	GBML10502-19
S32126	LSCL166	*Mercenaria mercenaria*	95% EtOH	EDTA008-21	*Mercenaria mercenaria*	100	GBML10502-19
