# Supplementary material for: Greater than pH 8: The pH dependence of EDTA as a preservative of high molecular weight DNA in biological samples
Source: PLoS One. 2023 Jan 23;18(1):e0280807. doi: 10.1371/journal.pone.0280807 (PMC9870144; doi:10.1371/journal.pone.0280807)
Supplement: S1 Raw images — DNA was extracted from (a) Alitta virens, (b) Faxonius virilis, (c) Homarus americanus, (d) Scomberomorus maculatus, and (e) Mercenaria mercenaria tissues after storage at room temperature for 12 months in 0.25 M EDTA pH 8 (lanes 1–10), 0.25 M EDTA pH 9 (lanes 11–20), 0.25 M EDTA pH 10 (lanes 21–30), and 95% ethanol (lanes 31–40). Fresh tissue extracts are shown in lanes 41–50. Lanes marked with an L contain 0.33 μL or 0.66 μL Quick Load Purple 1 kb Plus DNA Ladder (100 μg/mL; New England Biolabs; Ipswich, MA). Lanes marked with a red X indicate samples that were not recovered. Lanes marked with a black X indicate data that is not included in this investigation. Specimens are presented in the same order across all treatments. (PDF) [file pone.0280807.s003.pdf]

(a)

Alitta virens

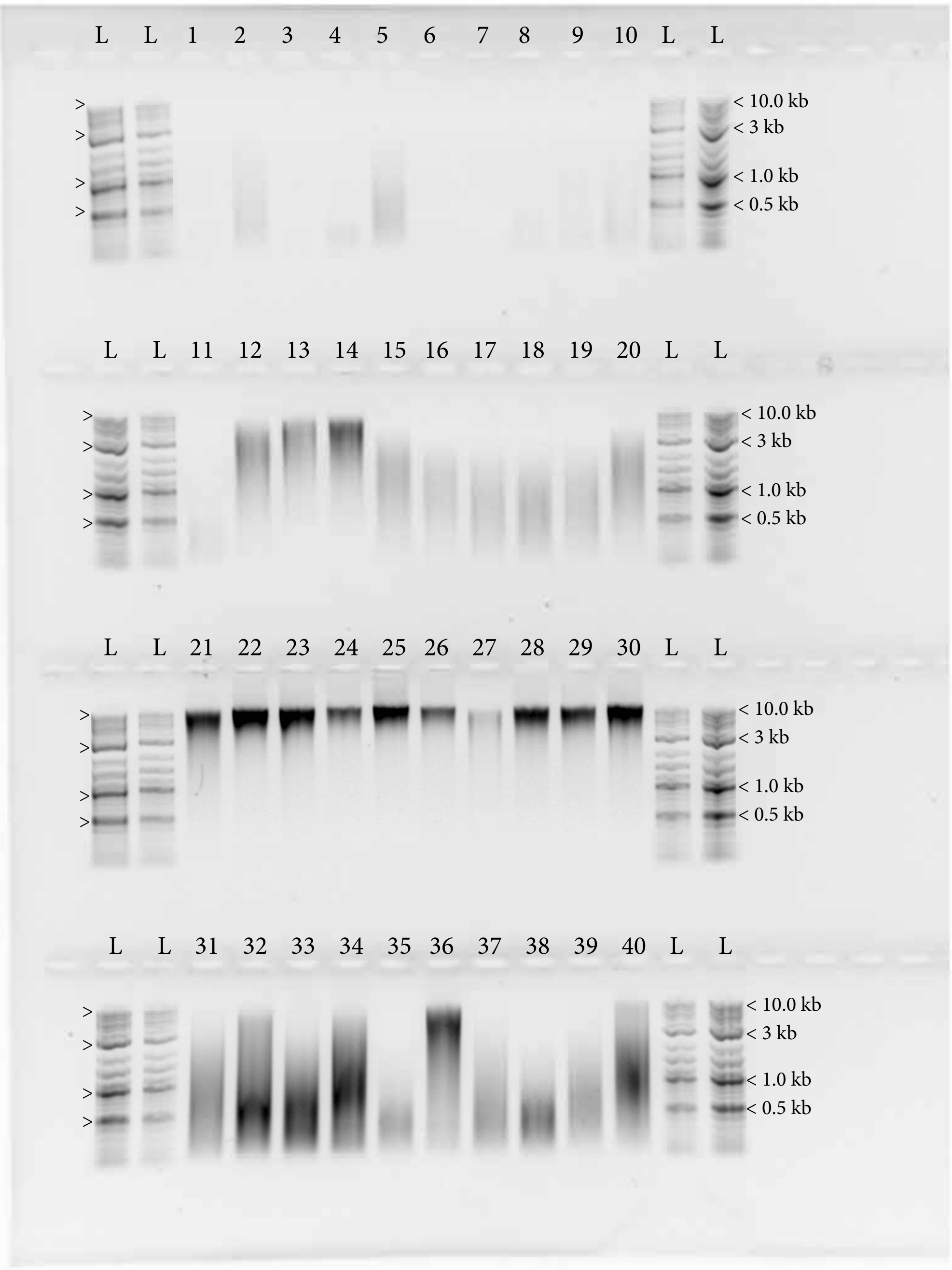

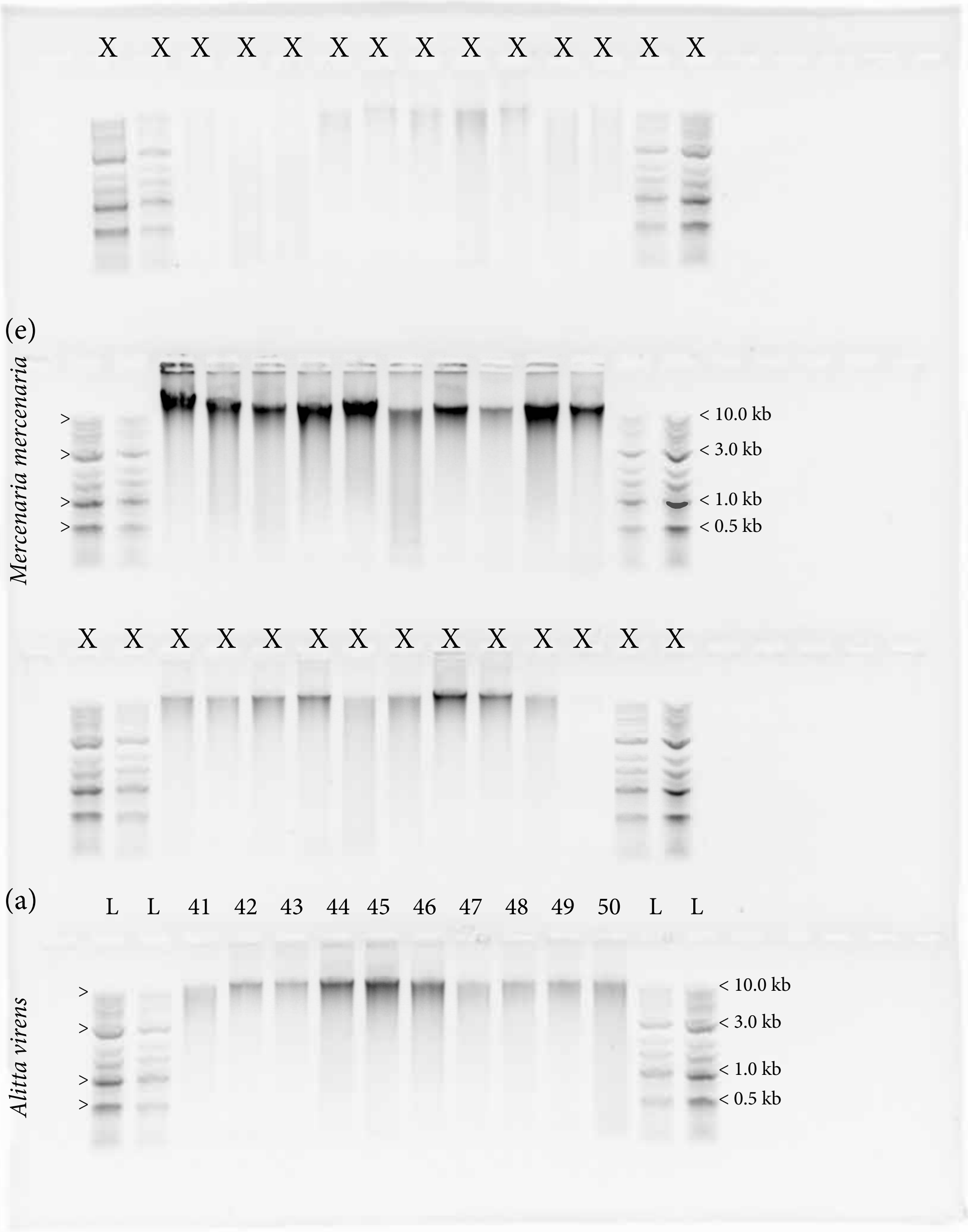
X X X X X X X X X X X X X X
(e)
Mercenaria mercenaria
< 10.0 kb
< 3.0 kb
< 1.0 kb
< 0.5 kb
X X X X X X X X X X X X X X
(a)
L L 41 42 43 44 45 46 47 48 49 50 L L
Alitta virens
< 10.0 kb
< 3.0 kb
< 1.0 kb
< 0.5 kb

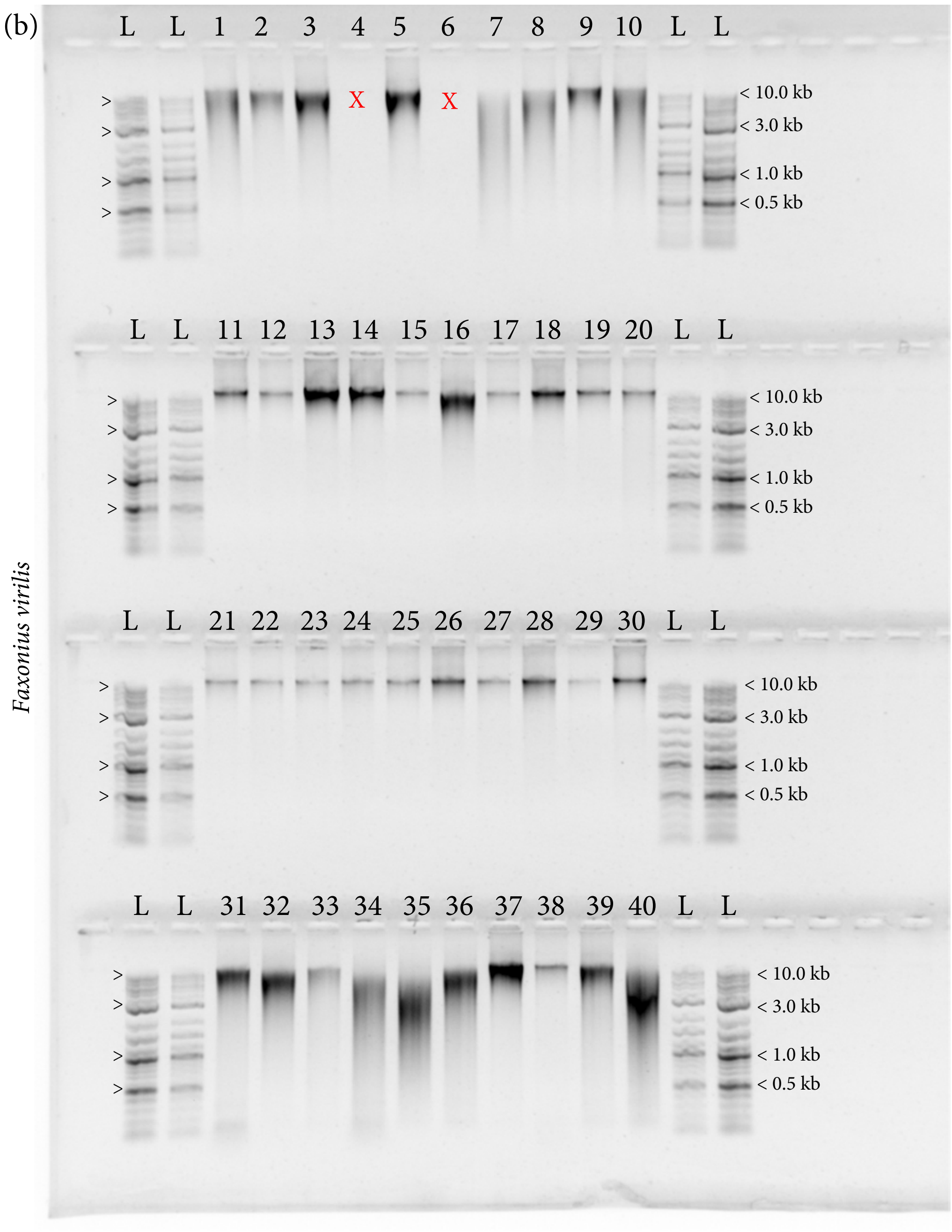
(b)
Faxonius virilis
L L 1 2 3 4 5 6 7 8 9 10 L L
L L 11 12 13 14 15 16 17 18 19 20 L L
L L 21 22 23 24 25 26 27 28 29 30 L L
L L 31 32 33 34 35 36 37 38 39 40 L L
X
X
< 10.0 kb
< 3.0 kb
< 1.0 kb
< 0.5 kb

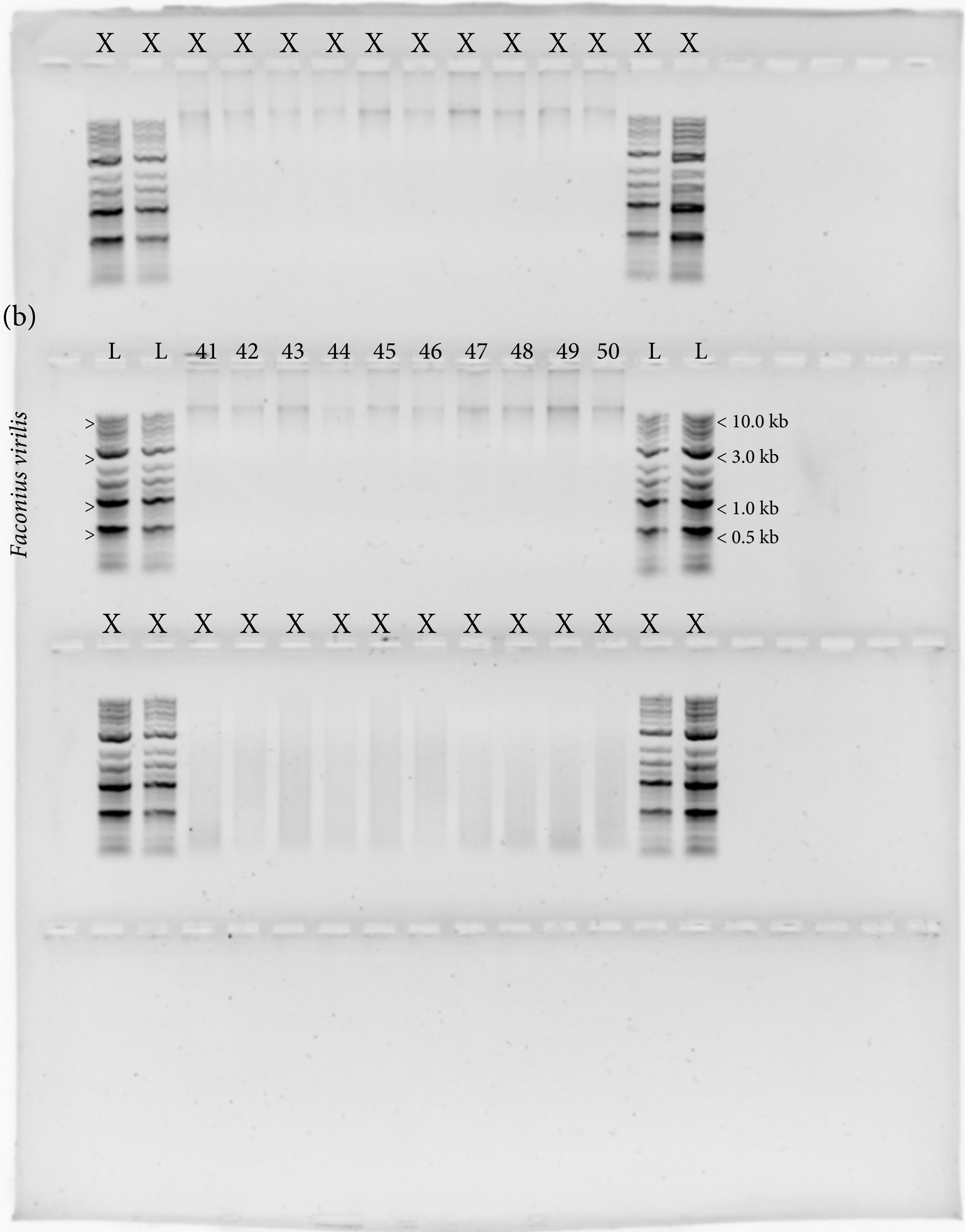
X X X X X X X X X X X X X X
(b)
L L 41 42 43 44 45 46 47 48 49 50 L L
Faconius virilis
< 10.0 kb
< 3.0 kb
< 1.0 kb
< 0.5 kb
X X X X X X X X X X X X X X

(c)

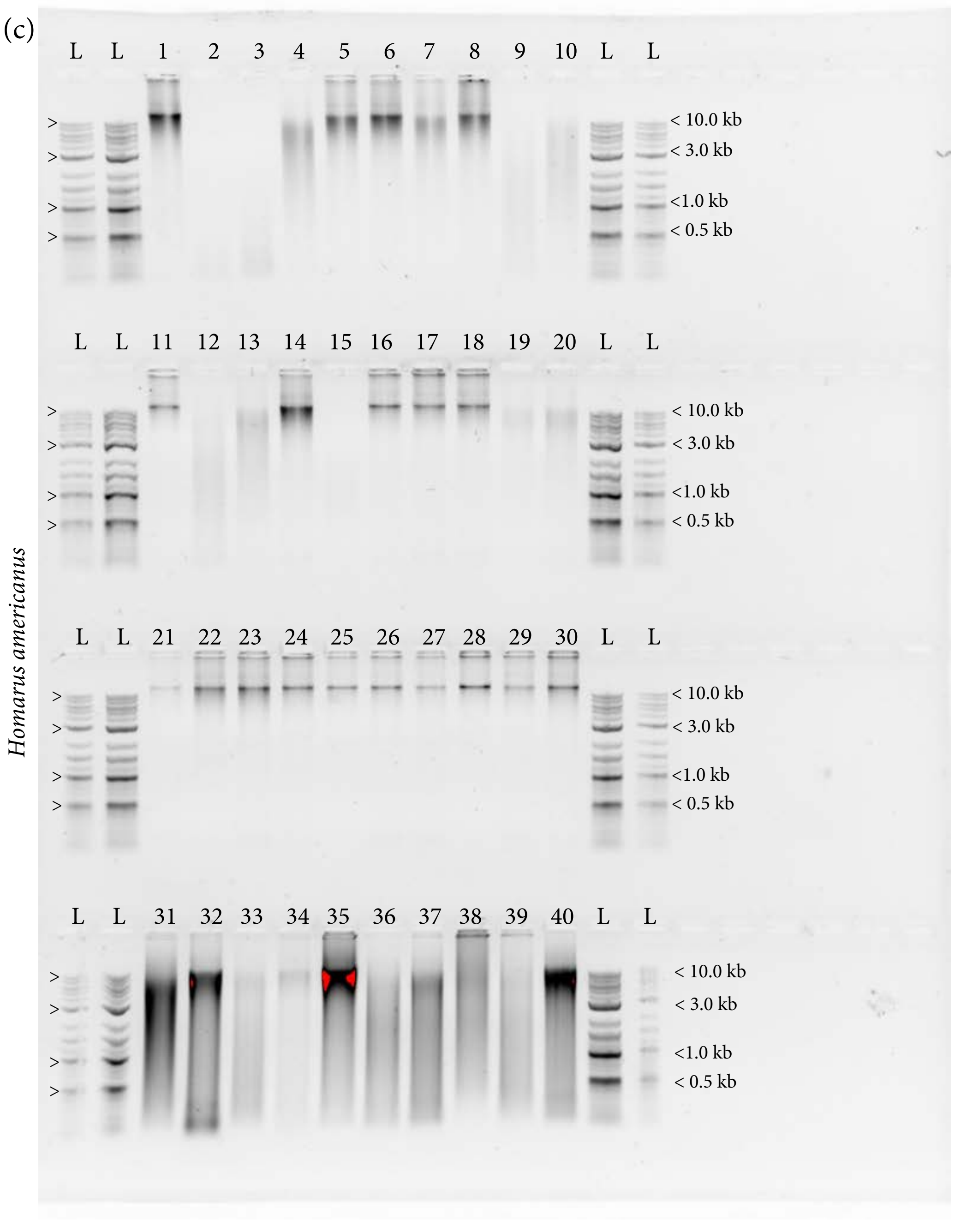

Homarus americanus
L L 1 2 3 4 5 6 7 8 9 10 L L
L L 11 12 13 14 15 16 17 18 19 20 L L
L L 21 22 23 24 25 26 27 28 29 30 L L
L L 31 32 33 34 35 36 37 38 39 40 L L
< 10.0 kb
< 3.0 kb
<1.0 kb
< 0.5 kb

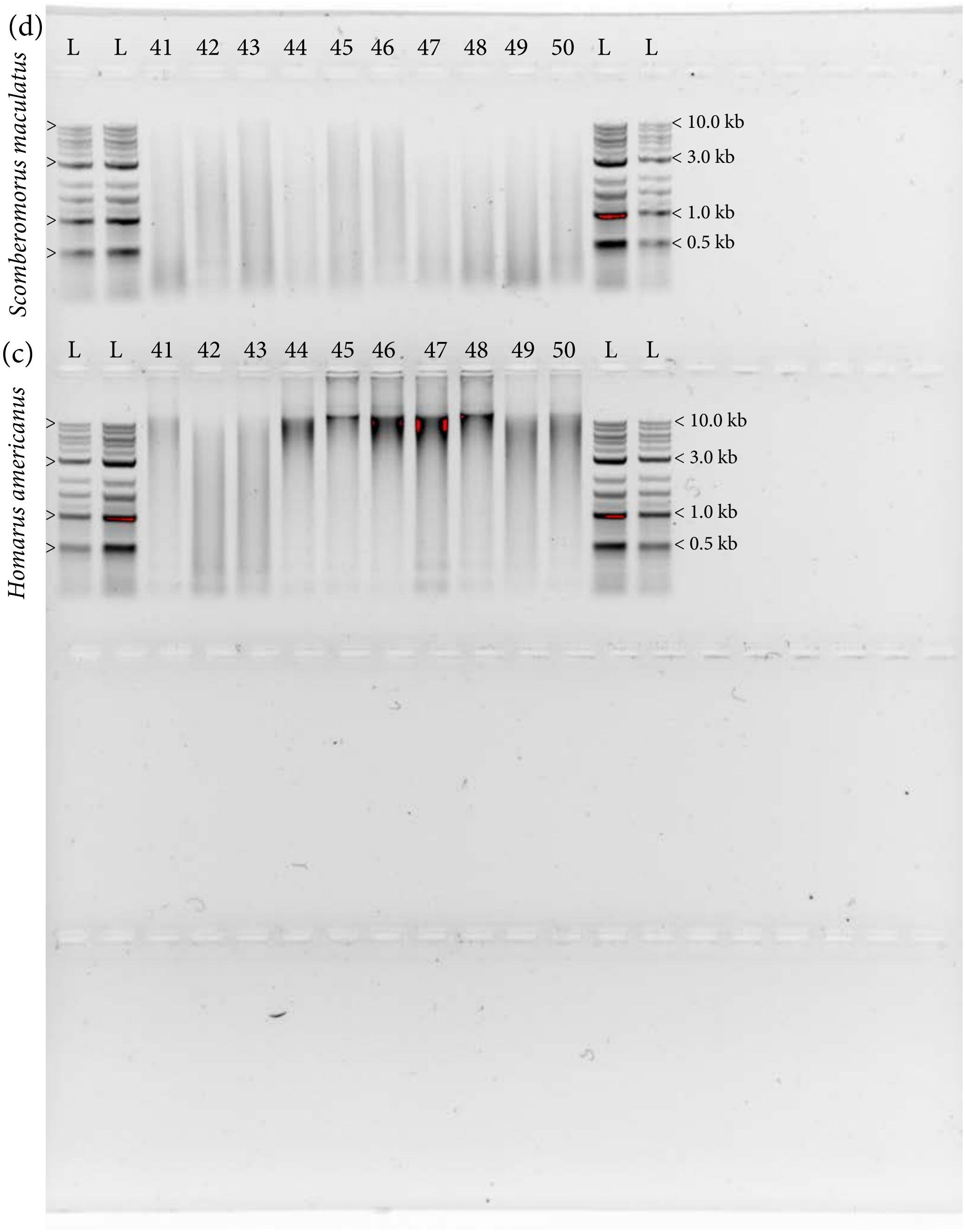
(d)
Scomberomorus maculatus
L L 41 42 43 44 45 46 47 48 49 50 L L
< 10.0 kb
< 3.0 kb
< 1.0 kb
< 0.5 kb
(c)
Homarus americanus
L L 41 42 43 44 45 46 47 48 49 50 L L
< 10.0 kb
< 3.0 kb
< 1.0 kb
< 0.5 kb

(d)

Scomberomorus maculatus

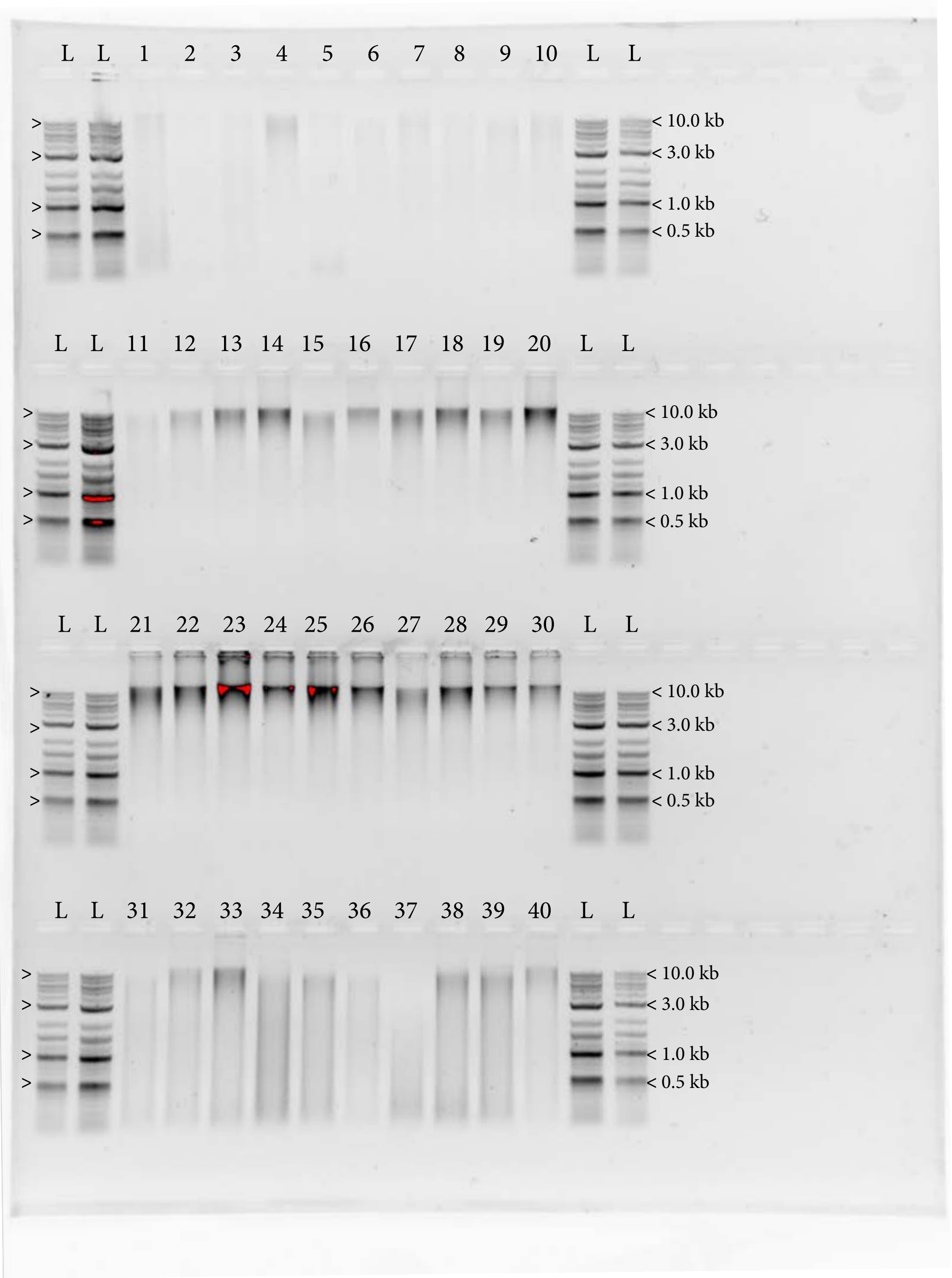

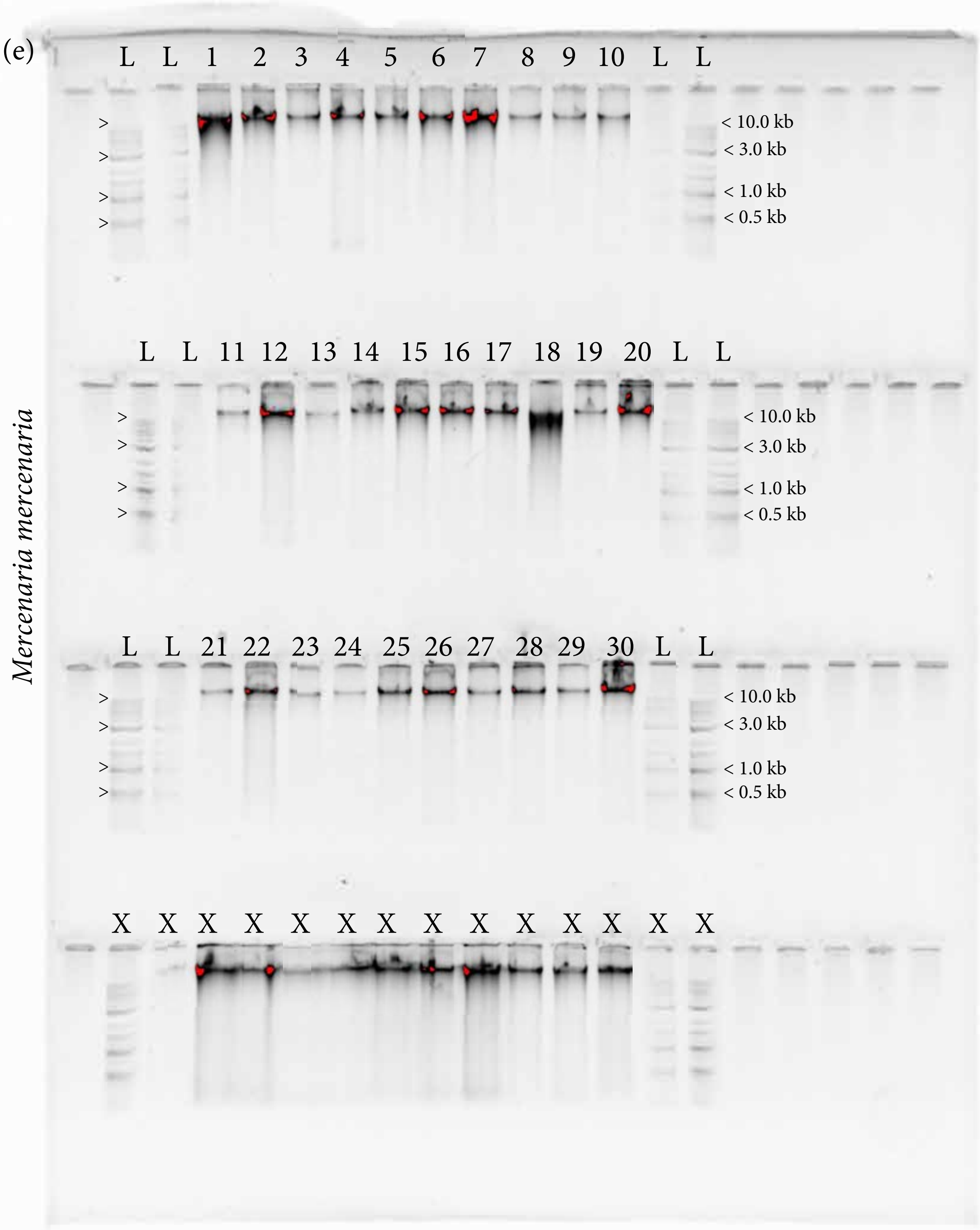
(e)
Mercenaria mercenaria
L L 1 2 3 4 5 6 7 8 9 10 L L
< 10.0 kb
< 3.0 kb
< 1.0 kb
< 0.5 kb
L L 11 12 13 14 15 16 17 18 19 20 L L
< 10.0 kb
< 3.0 kb
< 1.0 kb
< 0.5 kb
L L 21 22 23 24 25 26 27 28 29 30 L L
< 10.0 kb
< 3.0 kb
< 1.0 kb
< 0.5 kb
X X X X X X X X X X X X X X

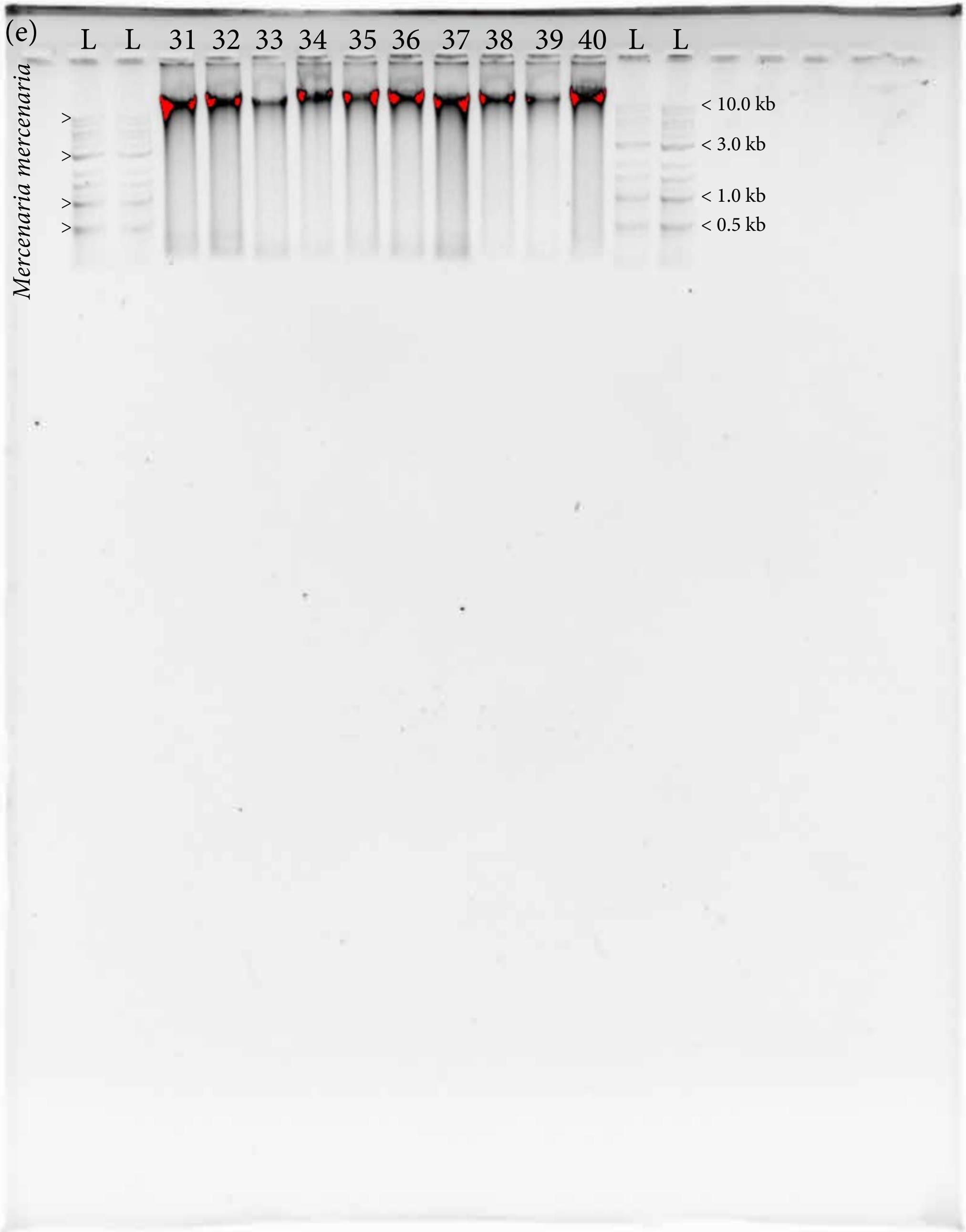
(e)
Mercenaria mercenaria
L L 31 32 33 34 35 36 37 38 39 40 L L
< 10.0 kb
< 3.0 kb
< 1.0 kb
< 0.5 kb
