# Supplementary material for: Greater than pH 8: The pH dependence of EDTA as a preservative of high molecular weight DNA in biological samples
Source: PLoS One. 2023 Jan 23;18(1):e0280807. doi: 10.1371/journal.pone.0280807 (PMC9870144; doi:10.1371/journal.pone.0280807)
Supplement: Attachment — Submitted filename: Response to Reviewers.docx [file pone.0280807.s004.docx]

**Response to Academic Editors and Reviewers**

PONE-D-22-14619

Greater than pH 8: the pH dependence of EDTA as a preservative of high molecular weight DNA in biological sample

**Response to Academic Editor**

Journal Requirements:

When submitting your revision, we need you to address these additional requirements.

1. Please ensure that your manuscript meets PLOS ONE's style requirements, including those for file naming. The PLOS ONE style templates can be found at

[https://journals.plos.org/plosone/s/file?id=wjVg/PLOSOne_formatting_sample_main_body.pdf](https://nam12.safelinks.protection.outlook.com/?url=https%3A%2F%2Fjournals.plos.org%2Fplosone%2Fs%2Ffile%3Fid%3DwjVg%2FPLOSOne_formatting_sample_main_body.pdf&data=05%7C01%7Cr.falco%40northeastern.edu%7Cfdb79f7e5b184884bb3f08dae377366f%7Ca8eec281aaa34daeac9b9a398b9215e7%7C0%7C0%7C638072398396571266%7CUnknown%7CTWFpbGZsb3d8eyJWIjoiMC4wLjAwMDAiLCJQIjoiV2luMzIiLCJBTiI6Ik1haWwiLCJXVCI6Mn0%3D%7C3000%7C%7C%7C&sdata=VGex8hvMLx5U7D%2BW1U1XoLWrBMhs35qoumAqqNbBNSI%3D&reserved=0)  and

[https://journals.plos.org/plosone/s/file?id=ba62/PLOSOne_formatting_sample_title_authors_affiliations.pdf](https://nam12.safelinks.protection.outlook.com/?url=https%3A%2F%2Fjournals.plos.org%2Fplosone%2Fs%2Ffile%3Fid%3Dba62%2FPLOSOne_formatting_sample_title_authors_affiliations.pdf&data=05%7C01%7Cr.falco%40northeastern.edu%7Cfdb79f7e5b184884bb3f08dae377366f%7Ca8eec281aaa34daeac9b9a398b9215e7%7C0%7C0%7C638072398396571266%7CUnknown%7CTWFpbGZsb3d8eyJWIjoiMC4wLjAwMDAiLCJQIjoiV2luMzIiLCJBTiI6Ik1haWwiLCJXVCI6Mn0%3D%7C3000%7C%7C%7C&sdata=18ugOX%2FBdtiw%2FRirTneC9n7RRW9P1spttUHeNJq7Krs%3D&reserved=0)

**Response:** We have renamed the Supporting Information files to match PLOS ONE’s requirements.

2. We note that the grant information you provided in the ‘Funding Information’ and ‘Financial Disclosure’ sections do not match.

When you resubmit, please ensure that you provide the correct grant numbers for the awards you received for your study in the ‘Funding Information’ section.

**Response:** We have updated the Funding Information and the Financial Disclosure sections to ensure that they agree. We have also included an additional funder, the Francis Goelet Chartable Lead Trust, which was accidentally omitted from the original submission.

3. PLOS ONE now requires that authors provide the original uncropped and unadjusted images underlying all blot or gel results reported in a submission’s figures or Supporting Information files. This policy and the journal’s other requirements for blot/gel reporting and figure preparation are described in detail at <https://journals.plos.org/plosone/s/figures#loc-blot-and-gel-reporting-requirements> and <https://journals.plos.org/plosone/s/figures#loc-preparing-figures-from-image-files>. When you submit your revised manuscript, please ensure that your figures adhere fully to these guidelines and provide the original underlying images for all blot or gel data reported in your submission. See the following link for instructions on providing the original image data: <https://journals.plos.org/plosone/s/figures#loc-original-images-for-blots-and-gels>.

In your cover letter, please note whether your blot/gel image data are in Supporting Information or posted at a public data repository, provide the repository URL if relevant, and provide specific details as to which raw blot/gel images, if any, are not available. Email us at [plosone@plos.org](mailto:plosone@plos.org)if you have any questions

**Response:** We have included the uncropped gel images in the Supporting Information and have labeled them to indicate the regions of the gels that have been cropped to create Fig 4. All the data used in this investigation has been submitted in the Supporting Information section of our manuscript and so have not been submitted to a public data repository.

4. In your Data Availability statement, you have not specified where the minimal data set underlying the results described in your manuscript can be found. PLOS defines a study's minimal data set as the underlying data used to reach the conclusions drawn in the manuscript and any additional data required to replicate the reported study findings in their entirety. All PLOS journals require that the minimal data set be made fully available. For more information about our data policy, please see <http://journals.plos.org/plosone/s/data-availability>.

Upon re-submitting your revised manuscript, please upload your study’s minimal underlying data set as either Supporting Information files or to a stable, public repository and include the relevant URLs, DOIs, or accession numbers within your revised cover letter. For a list of acceptable repositories, please see <http://journals.plos.org/plosone/s/data-availability#loc-recommended-repositories>. Any potentially identifying patient information must be fully anonymized.

Important: If there are ethical or legal restrictions to sharing your data publicly, please explain these restrictions in detail. Please see our guidelines for more information on what we consider unacceptable restrictions to publicly sharing data: <http://journals.plos.org/plosone/s/data-availability#loc-unacceptable-data-access-restrictions>. Note that it is not acceptable for the authors to be the sole named individuals responsible for ensuring data access.

We will update your Data Availability statement to reflect the information you provide in your cover letter.

**Response:** The complete minimal data set required for this investigation is reported in the Supporting Information section of the manuscript. This includes S1 Table entitled *Values for yield (μg), total normalized DNA yield (μg DNA/mg tissue), normalized high molecular weight DNA yield (nY; μg DNA/mg tissue), percent high molecular weight DNA recovered (%R), and COI PCR amplification success for each sample analyzed in this study*, S2 Table entitled *DNA barcoding*, and S1 Fig entitled *Raw agarose gel electrophoresis images for qualitative visualization of DNA fragment size distribution after 12 months*.

6. Please review your reference list to ensure that it is complete and correct. If you have cited papers that have been retracted, please include the rationale for doing so in the manuscript text, or remove these references and replace them with relevant current references. Any changes to the reference list should be mentioned in the rebuttal letter that accompanies your revised manuscript. If you need to cite a retracted article, indicate the article’s retracted status in the References list and also include a citation and full reference for the retraction notice.

**Response:** We have done so.

**Response to Reviewer Comments**

Reviewer #1: The manuscript is a great follow-up to a previous paper on DNA preservation using EDTA by the same laboratory (Sharpe et al., 2020, DESS deconstructed: Is EDTA solely responsible for protection of high molecular weight DNA in this common tissue preservative? PLOS ONE, <https://doi.org/10.1371/journal.pone.0237356>). In that study, the authors demonstrated that EDTA was the primary protector of high-molecular-weight DNA in the common preservative DESS. In this study they evaluate the effectiveness of EDTA as a DNA preservative at various pH’s. The results here are almost as striking and transformative as the previous study, notably so for difficult-to-extract taxa such as Alitta sp. Figure 4 is especially effective in demonstrating the utility of EDTA as a tissue preservative; the results shown in 4a are a bit astonishing.

This is an incredibly well-written and edited manuscript; I really have very few suggestions to make the body cleaner and more concise. The rare comments/questions are below.

Line 126, 127: Was the fresh tissue from the same individuals as the preserved tissue, which was extracted when the preserved tissues were put into storage? Alternatively, was the fresh tissue from new individuals, extracted with the preserved tissues after 12 months of storage? If the former, please briefly describe how the extracted DNA from fresh tissue was stored for those 12 months.

**Response**: We have modified the sentences to read as follows: *DNA was immediately extracted from the fifth tissue sample of the same individuals without preservation, hereafter referred to as fresh tissue. The DNA from these fresh tissue samples was visualized immediately after extraction by gel electrophoresis and then stored at -80°C for 12 months, at which time DNA from all treatments, including fresh tissue, were analyzed concomitantly* (lines 138-143).

Line 163, 164: Why are the spaces in the sequences for primers LCO1490_t1 and HCO2198_t2 placed where they are? I initially thought it was marking where the primer ended and the M13 tail started, but it does not. I’m not sure if a space is needed in the sequence, but if so, I would think the best place would be between the amplicon primer and the M13 tail.

**Response**: The extraneous spaces have been removed (lines 183 and 184).

Line 211: You mention taking Absorbance ratios, and include the results in S1 Table, but never mention them at any other time. Were there any significant differences among ratios? Did you run any statistical tests? I find that Absorbance ratios are not very useful and are highly inconsistent in their ability to determine DNA quality, but if they are included, I would possibly mention their results somewhere. Evaluating them with S1 Table is not especially useful.

**Response**: We agree with the reviewer’s assessment that the A260/A280 ratios reported in S1 Table are not very useful and that A260/A280 ratios are inconsistent in their ability to determine DNA quality. The A260/A280 ratio is an estimator of DNA purity, which was not examined in this investigation, and we used TapeStation data rather than A260 to estimate DNA concentration. For these reasons, we have removed A260/A280 ratios from the S1 Table and removed mention of them from the text, as suggested by the reviewer (removed from lines 178, 247, and 546).

Table 1: I would mention that the p-values are Bonferroni-corrected in the table description.

**Response**: The *p* values in Table 1 are not Bonferroni-corrected as they apply to single comparisons. However, we applied a Bonferroni correction to all post-hoc tests, e.g., in Figs 2 and 3. Here, the Bonferroni-corrected *p* value used for post-hoc tests is 0.005 rather than 0.05, reflecting the fact that ten pair-wise comparisons were made. We have corrected the legends of Figs 2 (line 263) and 3 (line 274) as follows: “*Within this figure, treatments bearing different lower-case letters are significantly different at the Bonferroni-adjusted p < 0.005*” to more accurately reflect the analysis performed. We have also clarified this point in the description of the statistical methods for parametric tests with the following text: “*If the data were normal, a one-way repeated measures ANOVA and a Bonferroni-corrected Tukey post-hoc test was performed with the “nlme” package*” (lines 237-239).

Additionally, in reviewing the statistical analyses we noticed minor error. In one taxon, *Faxonius virilis*, for %R, we had not applied a Bonferroni correction. We have now done so. As a result, %R for this taxon is not different between EDTA pH 10 and fresh tissue. We have modified the text to reflect this (lines 303, 372-375, 401, and 403). These changes do not affect our conclusion that the performance of EDTA as a preservative of HMW DNA in many tissues improves with increasing pH.

Reviewer #2: dear authors,

This is an important study for DNA isolation works but please clearly explain the aims of study in introduction section. What do you want to find from these results? whether these finding can be industrialized or would be just experimentally?

**Response**: We have added the following text to the introduction: “*Our aim in this investigation is to determine whether the performance of EDTA as a preservative of DNA in tissue can be improved by increasing pH. If so, this simple modification could be of value for research and commercial applications*” (lines 95-98).

**Additional Edits**

In reviewing the manuscript, we have made the following minor edits to improve clarity and readability.

**Line 21: Removed:** (235 words, limit 300)

**Line 45: Added:** —here defined as DNA fragments greater than 10 kb—

**Line 50: Modified:** adjusted to pH 7.5 to 8 **->** adjusted to a pH between 7.5 and 8

**Lines 85-86: Modified:** their capacity to bind metal ions **->** their capacity to do so

**Line 89: Removed:** invertebrate

**Line 90: Removed:** total

**Line 90: Modified:** These **->** We compare these

**Line 95: Removed:** were compared

**Line 111: Modified:** NaOH **->** sodium hydroxide

**Line 119: Modified:** , **->** :

**Line 150: Modified:** preservative **->** storage

**Lines 158-161: Modified:** Three μL (avg. 0.1071 ± 0.0995 μg DNA) of each DNA extract from A. virens, F. virilis, and M. mercenaria and 6 μL (avg. 0.0213 ± 0.0219 μg DNA) of each DNA extract from H. americanus and S. maculatus were loaded into a 16 cm horizonal slab gel **->** Three μL of each DNA extract from *A. virens*, *F. virilis,* and *M. mercenaria* (avg. 0.1071 ± 0.0995 μg DNA) and 6 μL of each DNA extract from *H. americanus* and *S. maculatus* (avg. 0.0213 ± 0.0219 μg DNA) were loaded into a 16 cm horizonal slab gel

**Lines 162-163: Modified:** The first and last two lanes of each gel were loaded with 0.33 μL and 0.66 μL of Quick Load Purple 1kb Plus DNA Ladder **->** The first and last lanes of each gel were loaded with either 0.33 μL or 0.66 μL of Quick Load Purple 1 kb Plus DNA Ladder

**Lines 176-177: Modified:** One μL (avg. 0.0243 ± 0.0296 μg DNA) of each DNA extract **->** One μL of each DNA extract (avg. 0.0243 ± 0.0296 μg DNA)

**Lines 218-224: Modified**: To estimate the quantity of HMW DNA, the quantity of low molecular weight DNA (150 bp to 10 kb) was subtracted from the total amount of DNA present in the sample, as determined by the Agilent Technologies TapeStation 2200 DNA Analyzer (Santa Clara, CA). We then determined the percent of HMW DNA recovered (%R) using the following formula **->** To estimate the quantity of HMW DNA, we first used the Agilent Technologies TapeStation 2200 DNA Analyzer (Santa Clara, CA) to evaluate the quantity of low molecular weight DNA (150 bp to 10 kb) and the total amount of DNA present in the sample. Then we calculated the quantity of HMW DNA for each sample by subtracting the low molecular weight DNA from the total amount of DNA present. Finally, we determined the percent of HMW DNA recovered (%R) using the following formula

**Line 284: Modified:** 1kb **->** 1 kb

**Line 320:** **Moved** “(Fig 4c)” from line 322 to line 320

**Lines 335-337: Modified:** Extracts of tissues stored in EDTA for all pH values tested yielded average values of %R significantly greater than those for 95% EtOH and fresh tissue **->** Extracts of tissues stored in EDTA pH 8, 9, and 10 yielded average %R values significantly greater than those for 95% EtOH and fresh tissue

**Line 367: Modified:** pH 8-10 **->** pH 8 to pH 10

**Line 371: Modified:** Specifically, after 12 months of room temperature storage, we observed significantly greater %R at pH 10 compared to pH 9 for two taxa, pH 10 compared to pH 8 for one taxon, and pH 9 compared to pH 8 for one taxon. Notably, in no case did %R decrease significantly with increasing pH **->** Specifically, after 12 months of room temperature storage, we observed significantly greater %R at pH 10 compared to pH 8 for these four taxa. Additionally, we observed significantly greater %R at pH 10 compared to pH 9 for two taxa and pH 9 compared to pH 8 for one taxon. Notably, in no case across all five taxa did %R decrease significantly with increasing pH

**Line 410: Added:** pH 10

**Line 414-415: Modified:** In our investigation, preservation of tissues in EDTA for all tested pH values had no adverse effect on the amplification of the COI gene **->** In our investigation, preservation of tissues in EDTA for pH values greater than 8 had no adverse effect on the amplification of the *COI* gene

**Line 560: Added:** database

**Line 560: Added:** Center

**Line 570: Modified:** 1kb **->** 1 kb
